# Accurate Attitude Estimation Using ARS under Conditions of Vehicle Movement Based on Disturbance Acceleration Adaptive Estimation and Correction

**DOI:** 10.3390/s16101716

**Published:** 2016-10-16

**Authors:** Li Xing, Yijun Hang, Zhi Xiong, Jianye Liu, Zhong Wan

**Affiliations:** College of Automation Engineering, Nanjing University of Aeronautics and Astronautics, Nanjing 211100, China; nuaaxl@nuaa.edu.cn (L.X.); hangyijun@nuaa.edu.cn (Y.H.); ljyac@nuaa.edu.cn (J.L.); wanzhong@nuaa.edu.cn (Z.W.)

**Keywords:** attitude reference system (ARS), attitude estimation, Kalman filter, disturbance acceleration, adaptive estimate and correction

## Abstract

This paper describes a disturbance acceleration adaptive estimate and correction approach for an attitude reference system (ARS) so as to improve the attitude estimate precision under vehicle movement conditions. The proposed approach depends on a Kalman filter, where the attitude error, the gyroscope zero offset error and the disturbance acceleration error are estimated. By switching the filter decay coefficient of the disturbance acceleration model in different acceleration modes, the disturbance acceleration is adaptively estimated and corrected, and then the attitude estimate precision is improved. The filter was tested in three different disturbance acceleration modes (non-acceleration, vibration-acceleration and sustained-acceleration mode, respectively) by digital simulation. Moreover, the proposed approach was tested in a kinematic vehicle experiment as well. Using the designed simulations and kinematic vehicle experiments, it has been shown that the disturbance acceleration of each mode can be accurately estimated and corrected. Moreover, compared with the complementary filter, the experimental results have explicitly demonstrated the proposed approach further improves the attitude estimate precision under vehicle movement conditions.

## 1. Introduction

An Attitude Reference System (ARS), which is widely applied to estimate the attitude of a vehicle [1,2] or the human body [3], is composed of orthogonally oriented sensors including a 3-axis gyroscope and 3-axis accelerometer. Typically speaking, the attitude is determined by integrating the gyroscope output. However, due to the existence of MEMS gyroscope zero offset error and bias instability, an accurate attitude estimation cannot be obtained by simple calculation using MEMS gyroscopes, which can only be utilized for a short time span to calculate attitude. As MEMS accelerometers are sensitive to the gravitational acceleration and can thus directly provide inclination information [4], the drifted attitude estimate error resulting from MEMS gyroscope data integration can be effectively corrected by fusing the output of a MEMS gyroscope with that of a MEMS accelerometer. Thus, a series of algorithms, which can be divided into three kinds, are studied for vehicle attitude estimation. One of the kinds are those algorithms based on vector observations [5,6,7,8,9], such as the TRIAD algorithm [6], QUEST algorithm [7], FOAM algorithm [8] and SVD algorithm [9]. These algorithms are deduced according to some specific physical or geometric significance. When these algorithms are used in ARS under vehicle movement conditions, the attitude estimate accuracy would be influenced by the MEMS accelerometer measurement errors [10]. In practice, multiple applications use complementary filters by fusing the output of a MEMS gyroscope and a MEMS accelerometer [11,12,13,14,15,16]. In earlier research, the attitude estimation system for an autonomous helicopter used a complementary filter obtained by fusing the output of a gyroscope and an inclinometer [15]. The attitude measuring principle of the inclinometer is similar to that of an accelerometer. The used of a complementary filter for determining the attitude was also applied in a fixed-wing UAV [16]. In these complementary filters, the output of the accelerometer or the inclinometer approximately equals the gravitational acceleration and does not consider the other component—the disturbance acceleration. As the output of the accelerometer is the sum of gravitational and inertial linear accelerations as mentioned in reference [17], and only the first component—the gravitational acceleration—is useful for ARS to determine the attitude, we name the second component—the inertial linear acceleration—the disturbance acceleration. Thus, when the ARS is stationary or moves with approximately uniform linear motion, the three components of the gravity acceleration are obtained and the disturbance acceleration is approximately zero, and then a ‘weak disturbance acceleration’ assumption is made in the filter. The filter fails, however, when vehicle movements are sufficiently strong that the accelerometer output no longer provides a good estimate of the gravitational direction [18]. When the vector observation algorithm or the complementary filter cannot take advantage of the data of the past and estimate sensor errors, this could be resolved by the state estimation algorithm [19,20,21,22,23,24,25,26]. A Kalman Filter is designed to estimate the orientation of human body segments by fusing gyroscope, accelerometer and magnetometer data [19]. In the state estimation algorithm, it could estimate not only the attitude, but also some uncertain error parameters (e.g., gyroscope offset errors), which reduces the impacts of uncertain error parameters on the attitude estimation. State variables of the state estimation algorithm contain attitude parameters as well as other variables for estimating attitude, and state equations are established in accordance with the kinematic differential equation. Moreover, in terms of the problems of nonlinearity in the ARS, there are a variety of nonlinear filter algorithms [22,23,24,25,26]. The Extended Kalman Filter (EKF) is the most widely-used nonlinear filter used in the ARS [23,24,25].

The state estimation algorithm based on Kalman Filter or nonlinear filters could estimate some uncertain parameters caused by some disturbance or sensor errors and improve the attitude precision to some extent. Nonetheless, how to effectively fuse the output of the gyroscope and accelerometer is still a problem when disturbance acceleration exists. In recent works, the threshold value of the disturbance acceleration to identify the acceleration level is utilized to isolate the disturbance acceleration. Based on the characteristics of gyros and accelerometers, the threshold values are extracted experimentally [14] or designed by fuzzy logic [26]. However, the threshold setting has a tight connection with the MEMS accelerometer performance. If the MEMS accelerometer has a large random noise, the threshold would be set at a bigger value. Thus, the disturbance acceleration could not be detected, and then the attitude and gyroscope zero offset would be estimated incorrectly. However, if the threshold is set as a small value, it would weaken the correcting effect of the accelerometer data on the gyroscope zero offset and the attitude. As a result, the attitude estimate would diverge. In other words, these existing attitude estimate algorithms directly use the gravity vector superposed the disturbance acceleration as the aid information for estimating the attitude, so their attitude estimate precision would be greatly influenced by the set threshold and the vehicle movement.

To cope with the abovementioned problems, this paper adds the disturbance acceleration error into the state vectors, and puts forward a disturbance acceleration adaptive estimate and correction approach based on a nine state vector Kalman filter that yields robust attitude estimate performance, even when the vehicle is accelerated in different modes—non-acceleration, vibration-acceleration and sustained-acceleration. In the filter, the attitude error, the gyroscope zero offset error and the disturbance acceleration error are all estimated. The disturbance acceleration is modeled as a first-order Markov Process, which is adaptively estimated by switching the filter decay coefficient for the disturbance acceleration Markov process. By estimating and correcting the disturbance acceleration, the observation accuracy of the gravity vector estimated by the accelerometer is effectively improved through deducting the disturbance acceleration from the accelerometer output, thus improving the roll and pitch estimate precision. Hence, the proposed approach produces robust estimates of roll and pitch. To verify the performance of the proposed approach, it was tested in three different acceleration modes (non-acceleration, vibration-acceleration and sustained-acceleration mode) by digital simulation. Kinematic vehicle experiments were also designed, and the estimation results were compared with the outputs of the on-board MEMS ARS (SBG Ellipse-N). By utilizing digital simulations and the kinematic vehicle experiments under conditions where disturbance acceleration exists, a significantly more accurate attitude estimate is obtained using the proposed approach compared with the complementary filter [14]. The comparison explicitly demonstrates that more accurate roll and pitch estimation performance can be achieved through the proposed disturbance acceleration adaptive estimate and correction approach based on a Kalman filter.

## 2. Attitude Estimation Based on the Disturbance Acceleration Adaptive Estimate and Correction

**Figure 1:**
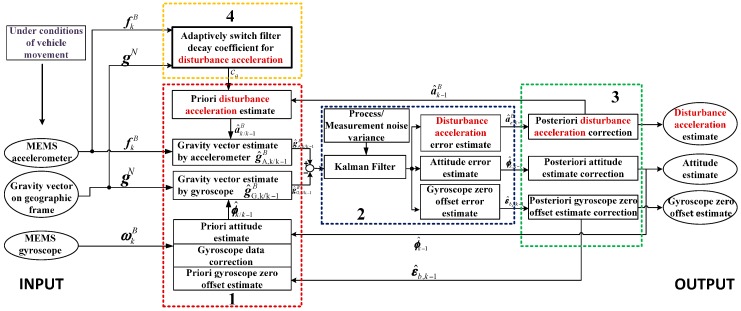
Framework of attitude estimation based on the disturbance acceleration adaptive estimate and correction.

Figure 1 refers to the attitude estimation framework based on the disturbance acceleration adaptive estimate and correction.

It includes four parts:

1. Calculation of the measurement error vector, that is the difference of gravity vector estimated by the gyroscope and accelerometer, respectively;
2. Estimation of attitude error, gyroscope zero offset error as well as the disturbance acceleration error;
3. Correction the estimate of the disturbance acceleration, the gyroscope zero offset and the attitude;
4. Adaptive adjustment of the filter decay coefficient of the disturbance acceleration model.

### 2.1. Model of the Error Process

In this paper, the state vector of the Kalman filter is designed as $x_{\varepsilon }=\left[\gamma_{\varepsilon }\;p_{\varepsilon }\;\varphi_{\varepsilon \;}\varepsilon_{b_{\varepsilon }x}\;\varepsilon_{b_{\varepsilon }y}\;\varepsilon_{b_{\varepsilon }z}\;a_{\varepsilon x}^{B}\;a_{\varepsilon y}^{B}\;a_{\varepsilon z}^{B}\right],$ where the first three entries represent Euler angle error ***θ****_ε_* including roll error *γ_ε_*, pitch error *p_ε_* and yaw error $\varphi_{\varepsilon }$. Additionally, $\varepsilon_{b_{\varepsilon }}={\left[\varepsilon_{b_{\varepsilon }x},\varepsilon_{b_{\varepsilon }y},\varepsilon_{b_{\varepsilon }z}\right]}^{T}$ represents the gyroscope zero offset error and $a_{\varepsilon }^{B}={\left[a_{\varepsilon x}^{B},a_{\varepsilon y}^{B},a_{\varepsilon z}^{B}\right]}^{T}$ represents the disturbance acceleration estimate error on body frame. In the indirect Kalman filter utilized for sensor fusion, the error process ***x****_ε_* updates recursively in Equation (1). Matrix ***A****_k_* and the noise component ***w****_k_* describe the propagation of the a priori error state vector. In this filter structure, the knowledge concerning previous errors is incorporated in the current state estimate. Consequently, there is no correlation between the a priori estimated errors between two time steps, meaning that the a priori errors do not depend on previous error states. Thus, the matrix ***A****_k_* is expressed as ***A****_k_* = **0**_9×9_. Moreover, the a priori estimate of the state vector ${\hat{x}}_{\varepsilon ,k/k-1}$ is abbreviated as Equation (2), while the a posteriori estimate of the state vector ${\hat{x}}_{\varepsilon ,k}$ is abbreviated as Equation (3), where ***Z****_ε,k_* refers to the measurement error:

(1) $$x_{\varepsilon ,k}=A_{k}x_{\varepsilon ,k-1}+w_{k}$$

(2) $${\hat{x}}_{\varepsilon ,k/k-1}=0$$

(3) $${\hat{x}}_{\varepsilon ,k}={\hat{x}}_{\varepsilon ,k/k-1}+K_{k}\left(Z_{\varepsilon ,k}-H_{k}{\hat{x}}_{\varepsilon ,k/k-1}\right)=K_{k}Z_{\varepsilon ,k}$$

### 2.2. Model of the Measurement Error Process

To calculate the a posteriori estimate of the state vector in Equation (3), the model of the measurement error process is defined. Meanwhile, the measurement vector is selected as the difference of the gravity vector estimate on the body frame (front-right-down) from the accelerometer and gyroscope, which is a 3 × 3 vector shown in Equation (4):

(4) $$Z_{k}={\hat{g}}_{A,k/k-1}^{B}-{\hat{g}}_{G,k/k-1}^{B}$$

(5) $${\hat{g}}_{A,k/k-1}^{B}=-f_{k}^{B}+{\hat{a}}_{k/k-1}^{B}$$

(6) $$a_{k}^{N}=c_{a}a_{k-1}^{N}+w_{a,k}$$

(7) $${\hat{a}}_{k/k-1}^{N}=c_{a}{\hat{a}}_{k-1}^{N}$$

(8) $${\hat{a}}_{k/k-1}^{B}={\hat{C}}_{N,k/k-1}^{B}{\hat{a}}_{k/k-1}^{N}=c_{a}\Delta C_{N}^{B}\left({\hat{\omega }}_{k/k-1}T\right){\hat{C}}_{N,k-1}^{B}{\hat{a}}_{k-1}^{N}\approx c_{a}\Delta C_{N}^{B}\left({\hat{\omega }}_{k/k-1}T\right){\hat{a}}_{k-1}^{B}\approx c_{a}{\hat{a}}_{k-1}^{B}$$

(9) $${\hat{a}}_{k}^{B}={\hat{a}}_{k-1}^{B}-{\hat{a}}_{\varepsilon ,k}^{B}$$

In Equation (4), ${\hat{g}}_{A,k/k-1}^{B}$ refers to the gravity vector estimate on body frame calculated from the accelerometer. The calculation process is shown in Equation (5), where $f_{k}^{B}$ denotes the output of the accelerometer at *t_k_* and ${\hat{a}}_{k/k-1}^{B}$ denotes the a priori estimate of the disturbance acceleration at *t_k_*. To isolate the disturbance acceleration and estimate the disturbance acceleration, the disturbance acceleration in the *N* (north-east-down) navigation frame is modelled as a first-order Markov process according to Equation (6) [27], where *c_a_* is the filter decay coefficient for the disturbance acceleration Markov process and ***w****_a,k_* is a zero mean white Gaussian noise process. Hence, the relationship of the a priori estimate of the disturbance acceleration (in the navigation frame *N*) and the a posteriori estimate (in the navigation frame *N*) of the previous iteration is shown in Equation (7). Then, the relationship of ${\hat{a}}_{k/k-1}^{B}$ and ${\hat{a}}_{k-1}^{B}$ is displayed in Equation (8) and ${\hat{a}}_{k-1}^{B}$ is calculated by Equation (9), which is the a posteriori estimate of the disturbance acceleration on body frame at *t_k__−1_*. In practice, the incremental orientation cosine matrix approximates $\Delta C_{N}^{B}\left({\hat{\omega }}_{k/k-1}T\right)\approx I_{3\times 3}$. ${\hat{\omega }}_{k/k-1}$ refers to the a priori angular velocity estimate; *T* refers to the Kalman Filter interval; ${\hat{C}}_{N,k/k-1}^{B}$ refers to the a priori estimate of the orientation cosine matrix at *t_k_*; ${\hat{C}}_{N,k-1}^{B}$ refers to the a posteriori estimate of the orientation cosine matrix at *t_k__−__1_*; ${\hat{a}}_{\varepsilon }^{B}={\left[{\hat{a}}_{\varepsilon x}^{B},{\hat{a}}_{\varepsilon y}^{B},{\hat{a}}_{\varepsilon z}^{B}\right]}^{T}$ refers to the a posteriori disturbance acceleration estimate error in the body frame. Substituting the calculated ${\hat{a}}_{k/k-1}^{B}$ into Equation (5), ${\hat{g}}_{A.k/k-1}^{B}$ is calculated.

(10) $${\hat{g}}_{G,k/k-1}^{B}={\hat{C}}_{N,k/k-1}^{B}g^{N}$$

In Equation (4), ${\hat{g}}_{G,k/k-1}^{B}$ represents the gravity vector estimate on the body frame calculated from the gyroscope. The calculation process can be shown in Equation (10), where ***g****^N^* is the gravity vector in the navigation frame *N*. ${\hat{C}}_{N,k/k-1}^{B}$ is calculated by the a priori estimate of the quaternion ${\hat{q}}_{k/k-1}$.

(11) $$Z_{\varepsilon ,k}={\hat{g}}_{A,k/k-1}^{B}-{\hat{g}}_{G,k/k-1}^{B}=H_{k}{\hat{x}}_{\varepsilon ,k/k-1}+v_{k}=H_{k}\left(\begin{array}{c} \theta_{\varepsilon }\\ \varepsilon_{b_{\varepsilon }}\\ a_{\varepsilon }^{B} \end{array}\right)+v_{k}$$

(12) $$H_{k}=\left(\begin{array}{ccccccccc} 0 & {\hat{g}}_{Gz,k/k-1}^{B} & -{\hat{g}}_{Gy,k/k-1}^{B} & 0 & -{\hat{g}}_{Gz,k/k-1}^{B}T & {\hat{g}}_{Gy,k/k-1}^{B}T & 1 & 0 & 0\\ -{\hat{g}}_{Gz,k/k-1}^{B} & 0 & {\hat{g}}_{Gx,k/k-1}^{B} & {\hat{g}}_{Gz,k/k-1}^{B}T & 0 & -{\hat{g}}_{Gx,k/k-1}^{B}T & 0 & 1 & 0\\ {\hat{g}}_{Gy,k/k-1}^{B} & -{\hat{g}}_{Gx,k/k-1}^{B} & 0 & -{\hat{g}}_{Gy,k/k-1}^{B}T & {\hat{g}}_{Gx,k/k-1}^{B}T & 0 & 0 & 0 & 1 \end{array}\right)$$

Equation (11) gives the relationship between the measurement vector and the state vector, and the measurement matrix ***H****_k_* can be expressed as Equation (12).

### 2.3. State Errors Estimate and Correction Process

Combining Equations (2) and (11), Equation (13) gives the estimate process of the attitude error, the gyroscope zero offset error and the disturbance acceleration error on body frame. As shown in (13), the a priori 9 × 9 error covariance matrix ***P****_k/k__−_*_1_ is equal to the state noise matrix ***Q****_k_* in the filter model, which is different from the conventional Kalman filter. In Equation (13), expanding ***Q****_k_* = ***P****_k/k__−_*_1_ gives Equation (14). Through further deduction, we can find that ***Q****_k_* could be updated for the next iteration as a function of the a posteriori covariance matrix ***P****_k__−_*_1_ shown in Equation (15), in which $Q_{w\varepsilon_{b}}I_{3\times 3}$ denotes the noise matrix of the gyroscope zero offset white noise, *Q_wb_**I***_3__×__3_ denotes the noise matrix of the gyroscope white noise and *Q_wa_**I***_3__×__3_ denotes the noise matrix of the disturbance acceleration white noise shown in Equation (16). From Equation (14), we can see that the relationship among the attitude error, the gyroscope zero offset error and the disturbance acceleration error is reflected in the updated process of ***Q****_k_*, so ***A****_k_* = **0**_9__×__9_ is reasonable, although it is different from the conventional Kalman filter. Equation (17) gives the calculation process of the measurement noise matrix ***R****_k_*, where *Q_vA_**I***_3__×__3_ refers to the noise matrix of the accelerometer white noise.

(13) $$\left\{ \begin{array}{l} {\hat{x}}_{\varepsilon ,k/k-1}=0\\ P_{k/k-1}=A_{k}P_{k-1}A_{k}^{T}+Q_{k}=Q_{k}\\ K_{k}=P_{k/k-1}H_{k}^{T}{\left(H_{k}P_{k/k-1}H_{k}^{T}+R_{k}\right)}^{-1}=Q_{k}H_{k}^{T}{\left(H_{k}Q_{k}H_{k}^{T}+R_{k}\right)}^{-1}\\ {\hat{x}}_{\varepsilon ,k}=K_{k}Z_{\varepsilon ,k}\\ P_{k}=\left(I-K_{k}H_{k}\right)P_{k/k-1}=\left(I-K_{k}H_{k}\right)Q_{k} \end{array}\right.$$

(14) $$Q_{k}=P_{k/k-1}=E\left[\begin{array}{ccc} {\hat{\theta }}_{\varepsilon ,k/k-1}{\left({\hat{\theta }}_{\varepsilon ,k/k-1}\right)}^{T} & {\hat{\theta }}_{\varepsilon ,k/k-1}{\left({\hat{\varepsilon }}_{b_{\varepsilon },k/k-1}\right)}^{T} & {\hat{\theta }}_{\varepsilon ,k/k-1}{\left(a_{\varepsilon ,k/k-1}^{B}\right)}^{T}\\ {\hat{\varepsilon }}_{b_{\varepsilon },k/k-1}{\left({\hat{\theta }}_{\varepsilon ,k/k-1}\right)}^{T} & {\hat{\varepsilon }}_{b_{\varepsilon },k/k-1}{\left({\hat{\varepsilon }}_{b_{\varepsilon },k/k-1}\right)}^{T} & {\hat{\varepsilon }}_{b_{\varepsilon },k/k-1}{\left(a_{\varepsilon ,k/k-1}^{B}\right)}^{T}\\ a_{\varepsilon ,k/k-1}^{B}{\left({\hat{\theta }}_{\varepsilon ,k/k-1}\right)}^{T} & a_{\varepsilon ,k/k-1}^{B}{\left({\hat{\varepsilon }}_{b_{\varepsilon },k/k-1}\right)}^{T} & a_{\varepsilon ,k/k-1}^{B}{\left(a_{\varepsilon ,k/k-1}^{B}\right)}^{T} \end{array}\right]$$

(15) $$\begin{array}{l} Q_{k}=f(P_{k-1})=\left[\begin{array}{ccc} Q_{1k} & Q_{2k} & 0_{3\times 3}\\ Q_{2k} & Q_{3k} & 0_{3\times 3}\\ 0_{3\times 3} & 0_{3\times 3} & Q_{4k} \end{array}\right]\\ \{\begin{array}{c} \begin{array}{l} Q_{1k}=P_{k-1}[1,3:1,3]+T^{2}\left[P_{k-1}[4,6:4,6]+\left(Q_{w\varepsilon_{b}}+Q_{wb}\right)I_{3\times 3}\right]\\ Q_{2k}=-T(P_{k-1}[1,3:4,6]+Q_{w\varepsilon_{b}}I_{3\times 3})\\ Q_{3k}=P_{k-1}[4,6:4,6]+Q_{w\varepsilon_{b}}I_{3\times 3}\\ Q_{4k}=c_{a}P_{k-1}[7,9:7,9]+Q_{wa}I_{3\times 3} \end{array} \end{array} \end{array}$$

(16) $$\{\begin{array}{c} \begin{array}{l} Q_{w\varepsilon_{b}}I_{3\times 3}=E\left[w_{\varepsilon_{b,k}}{\left(w_{\varepsilon_{b,k}}\right)}^{T}\right]\\ Q_{wb}I_{3\times 3}=E\left[w_{b,k}{\left(w_{b,k}\right)}^{T}\right]\\ Q_{vA}I_{3\times 3}=E\left[v_{A,k}{\left(v_{A,k}\right)}^{T}\right]\\ Q_{wa}I_{3\times 3}=E\left[w_{a,k}{\left(w_{a,k}\right)}^{T}\right] \end{array} \end{array}$$

(17) $$R_{k}=\left[Q_{vA}+Q_{wa}+T^{2}\left(Q_{w\varepsilon_{b}}+Q_{wb}\right)\right]I_{3\times 3}$$

By estimating state errors, states in relation to attitude estimate are corrected further, so as to improve the attitude estimate precision in ARS. State corrections include the attitude correction (the orientation cosine matrix correction or the quaternion correction), the output of gyroscope correction as well as the disturbance acceleration correction on body frame. The state correction process is given by Equations (18) and (19). In MEMS ARS, the velocity and the position cannot be calculated. Beyond that, the a priori MEMS gyroscope correction estimate ${\hat{\omega }}_{k/k-1}^{B}\gg {\hat{C}}_{N,k/k-1}^{B}\left({\hat{\omega }}_{IE,k/k-1}^{N}+{\hat{\omega }}_{EN,k/k-1}^{N}\right)$, where ${\hat{\omega }}_{IE,k/k-1}^{N}$ denotes the a priori estimate of the earth’s rotation angular in the navigation frame and ${\hat{\omega }}_{EN,k/k-1}^{N}$ denotes the a priori estimate of rotation angular from the navigation frame to the geocentric Earth frame. Thus, the a priori angular velocity estimate ${\hat{\omega }}_{k/k-1}$ approximately equals the a priori MEMS gyroscope correction estimate ${\hat{\omega }}_{k/k-1}^{B}$.

(18) $$\begin{array}{l} {\hat{q}}_{k}={\hat{q}}_{k/k-1}\Delta q\left(-{\hat{\phi }}_{\varepsilon ,k}\right)\\ {\hat{q}}_{k/k-1}={\hat{q}}_{k-1}\Delta q\left({\hat{\omega }}_{k/k-1}T\right)\\ {\hat{\omega }}_{k/k-1}={\hat{\omega }}_{k/k-1}^{B}-{\hat{C}}_{N,k/k-1}^{B}({\hat{\omega }}_{IE,k/k-1}^{N}+{\hat{\omega }}_{EN,k/k-1}^{N})\approx {\hat{\omega }}_{k/k-1}^{B}\\ {\hat{\omega }}_{k/k-1}^{B}=\omega_{k}^{B}-{\hat{\varepsilon }}_{b,k/k-1}\\ {\hat{\varepsilon }}_{b,k/k-1}={\hat{\varepsilon }}_{b,k-1}\\ {\hat{\varepsilon }}_{b,k}={\hat{\varepsilon }}_{b,k/k-1}-{\hat{\varepsilon }}_{b_{\varepsilon },k}={\hat{\varepsilon }}_{b,k-1}-{\hat{\varepsilon }}_{b_{\varepsilon },k} \end{array}$$

Equation (18) gives the process of the attitude correction and the gyroscope output correction, where ${\hat{q}}_{k/k-1}$ is the a priori estimate of the attitude quaternion, while ${\hat{q}}_{k}$ is the a posteriori estimate of the attitude quaternion. ${\hat{\omega }}_{k}^{B}$ represents the MEMS gyroscope output, ${\hat{\varepsilon }}_{b.k}$ represents the a posteriori estimate of the gyroscope zero offset, and ${\hat{\varepsilon }}_{b,k/k-1}$ represents the a priori estimate of the gyroscope zero offset.

(19) $$\begin{array}{l} {\hat{a}}_{k}^{B}={\hat{a}}_{k/k-1}^{B}-{\hat{a}}_{\varepsilon ,k}^{B}\\ {\hat{a}}_{k/k-1}^{B}=c_{a}{\hat{a}}_{k-1}^{B} \end{array}$$

Equation (19) gives the process of the disturbance acceleration correction on body frame. The measurement error at *t_k_* is computed by respectively substituting Equations (18) and (19) into Equations (5) and (10). Then, utilizing the computed measurement error and corrected state errors, the attitude estimate could be corrected by the filter given in Equation (13).

### 2.4. Adaptive Estimation of Disturbance Acceleration

If the disturbance acceleration was not estimated, the gravity vector estimate on body frame ${\hat{g}}_{A,k/k-1}^{B}$ would equal the accelerometer output as shown in Equation (5), which could affect the observation accuracy of the gravity vector. The observation error would be distributed to the attitude error as well as gyroscope zero offset error, as shown in Equation (11), and then influence the attitude estimate accuracy. To solve the problem, the complementary filter weakens the disturbance acceleration effect by tuning the proportional and integral coefficients [14]. Nevertheless, the approach could not effectively improve the precision of the attitude estimate. Because it is hard to set the appropriate proportional and integral coefficients to balance disturbance acceleration influence and gyro offset error influence on the attitude estimation under conditions of vehicle movement. In the conventional Kalman filter without the acceleration estimate [23], when there is the disturbance acceleration, the measurement noise matrix *R* should be increased to reduce the filter gain and decrease the impact of innovation vector (the attitude estimate difference between accelerometer and gyroscope) on the attitude error. The approach is similar to the complementary filter, which could not effectively weaken the disturbance acceleration influence, especially when the vehicle accelerates for an extended period. Since the two filters did not model the disturbance acceleration for estimating, the observation accuracy of the gravity vector could not be effectively improved by deducting the disturbance acceleration from the accelerometer output, and meanwhile the disturbance acceleration would still disturb the attitude estimate. 

In the disturbance acceleration estimate model in Equation (19), the decay filter coefficient *c_a_* is proportional to the correlation time of the first-order Markov process as shown in Equation (20) and 0 < *c_a_* < 1. If the disturbance acceleration changes in a low frequency, the correlation time is short and *c_a_* would be set at a larger value. If the disturbance acceleration changes at a high frequency, which is similar to vibration conditions, *c_a_* would be set as a smaller value. By switching *c_a_*, the disturbance acceleration estimate would not only accord with the actual linear acceleration but also improve the observation accuracy of the gravity vector. In this way, it would improve the attitude estimate precision. As the measurement of the disturbance acceleration frequency is impractical, *c_a_* is adaptively switched according to the norm of the disturbance acceleration *a_k_* in the actual vehicle movement:

(20) $$a_{k}^{N}=c_{a}a_{k-1}^{N}+w_{a,k}=e^{-\frac{1}{\tau }}a_{k-1}^{N}+w_{a,k}$$

In the proposed approach, the value of the filter decay coefficient *c_a_* is switched in accordance with the acceleration modes (non-acceleration, vibration-acceleration and sustained-acceleration mode) determined by the norm of the disturbance acceleration *a_k_*, defined as Equation (21). In Equation (21) *g* refers to the norm of the gravity vector. Based on the characteristics of accelerometers, the threshold value *a_threshold_* to identify the acceleration mode is extracted experimentally. In the proposed approach, *a_threshold_* refers to the accelerometer noise mean-square deviation when the vehicle is static. What’s more, the computation process is shown in Equation (22), in which *t_total_static_* signifies the total static time, while *T_a_* signifies the sample period of the accelerometer:

(21) $$\{\begin{array}{c} a_{k}=\left|\sqrt{f_{x}^{B}{\left(k\right)}^{2}+f_{y}^{B}{\left(k\right)}^{2}+f_{z}^{B}{\left(k\right)}^{2}}-g\right|\\ g=\sqrt{{\left(g_{x}^{N}\right)}^{2}+{\left(g_{y}^{N}\right)}^{2}+{\left(g_{z}^{N}\right)}^{2}} \end{array}$$

(22) $$\{\begin{array}{c} a_{threshold}=\sqrt{\frac{\sum_{k=1}^{n}{\left(\sqrt{f_{x}^{B}{\left(k\right)}^{2}+f_{y}^{B}{\left(k\right)}^{2}+f_{z}^{B}{\left(k\right)}^{2}}-g\right)}^{2}}{n}}\\ n=\frac{t_{total\_static}}{T_{a}} \end{array}$$

The switching logic for the filter decay coefficient *c_a_* has the following scenarios:

1. Non-acceleration mode: In this mode, the paper assumes that *a_k_* < 3*a_threshold_*. Owing to the little amplitude of the disturbance acceleration, and the acceleration estimate should decay in a short period in the model, namely *τ* in Equation (20), should be set as a small value. Thus, the filter decay coefficient is set as 0 < *c_a_* ≤ 0.5.
2. Vibration-acceleration mode: In this mode, the paper assumes that 3*a_threshold_* ≤ *a_k_* < 50*a_threshold_*. With respect to the changes of the disturbance acceleration, the decay period *τ* of the acceleration estimate should be set longer than for mode 1. The filter decay coefficient is set as $c_{a}=0.5\times \frac{a_{k}-3a_{threshold}}{50a_{threshold}-3a_{threshold}}+0.5.$ As shown in the formula, *c_a_* is adaptively adjusted with respect to *a_k_*.
3. Sustained-acceleration mode: In this mode, the paper assumes that *a_k_* ≥ 50*a_threshold_*. As the vehicle is subject to strong and sustained acceleration, the filter decay coefficient *c_a_* is set as 1.0.

**Figure 2:**
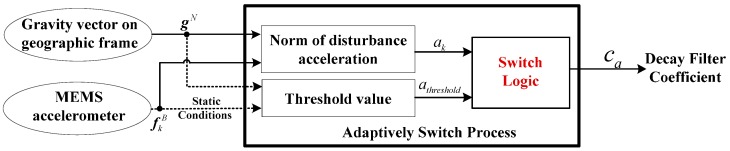
Adaptive switch process of filter decay coefficient *c_a_*.

The adaptive switch process of filter decay coefficient *c_a_* is shown in Figure 2.

## 3. Experiments and Results

### 3.1. Digital Simulation Experiments and Results

**Figure 3:**
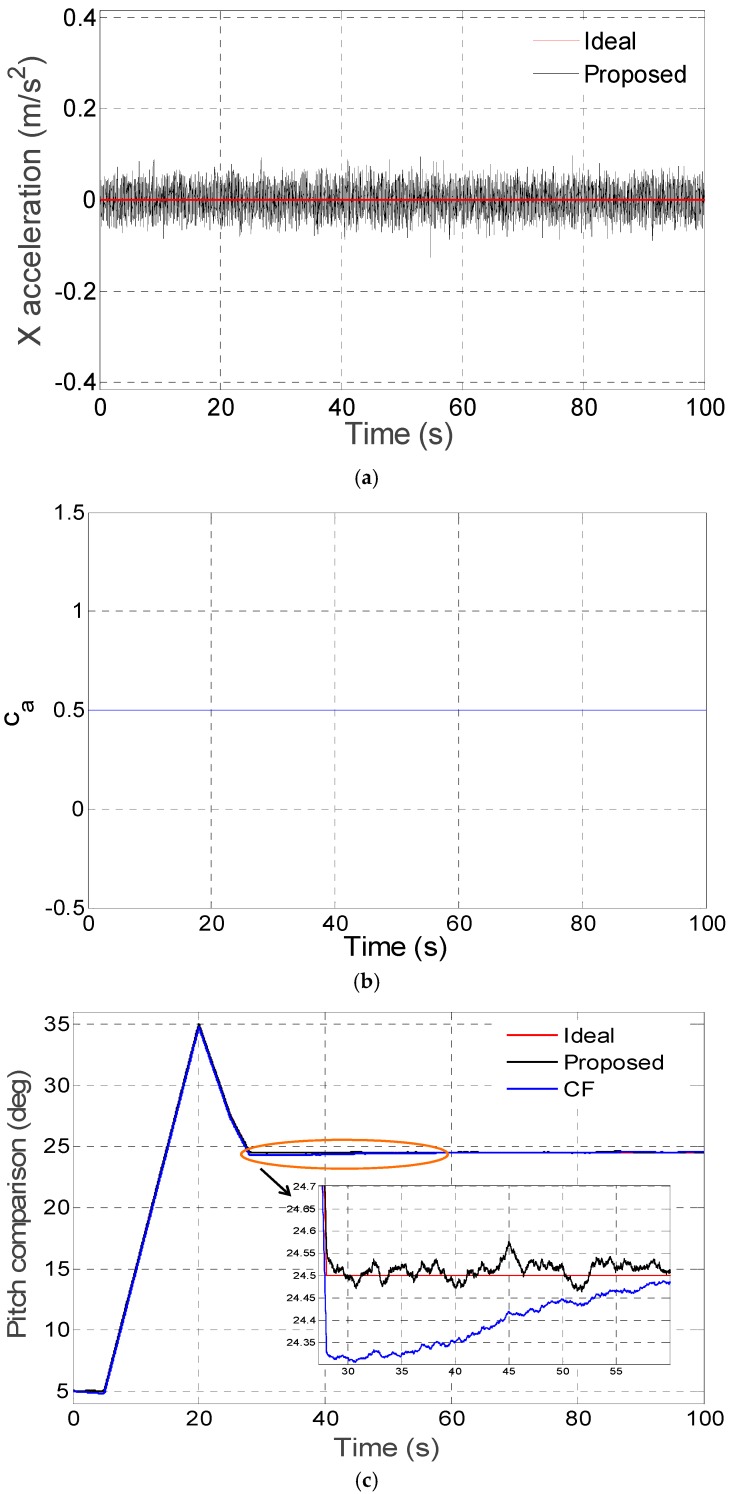
(**a**) X axis disturbance acceleration estimation on body frame in non-acceleration mode; (**b**) Adaptive switch for *c_a_* in non-acceleration mode; (**c**) Pitch estimate comparison in non-acceleration mode.

**Figure 4:**
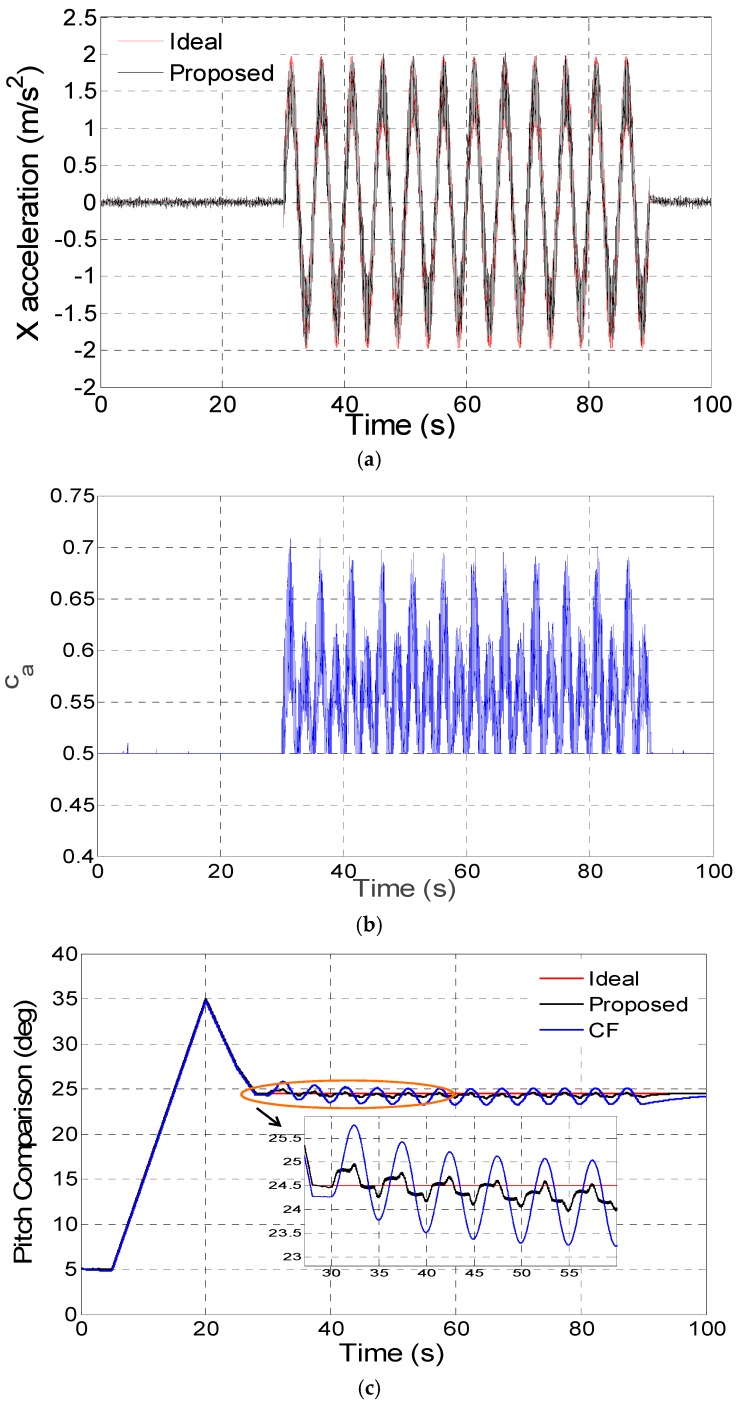
(**a**) X axis disturbance acceleration estimation on body frame in vibration-acceleration mode; (**b**) Adaptive switch for *c_a_* in vibration-acceleration mode; (**c**) Pitch estimate comparison in vibration-acceleration mode.

**Figure 5:**
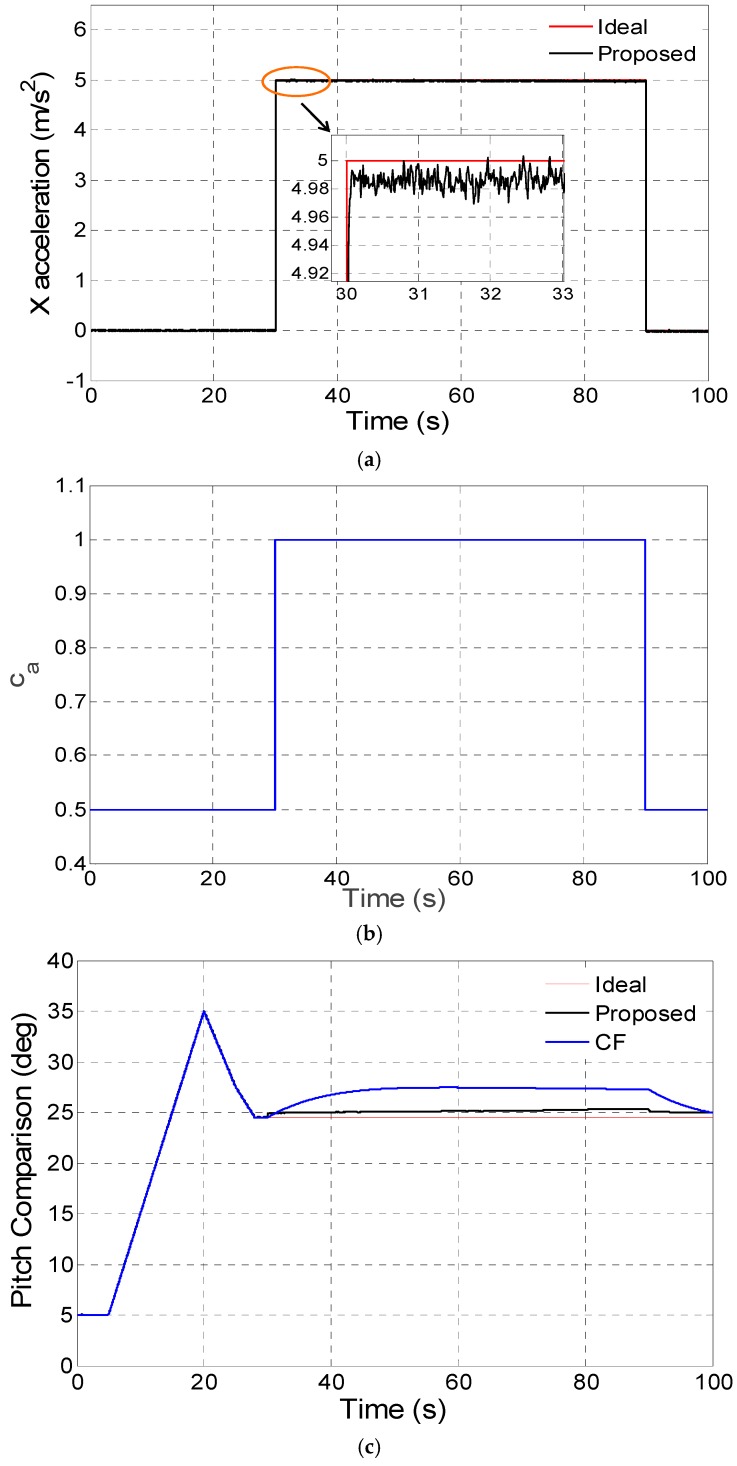
(**a**) X axis disturbance acceleration estimation on body frame in sustained-acceleration mode; (**b**) Adaptive switch for *c_a_* in sustained-acceleration mode; (**c**) Pitch estimate comparison in sustained-acceleration mode.

The proposed disturbance acceleration adaptive estimate and correction approach based on switching the filter decay coefficient *c_a_* in three different disturbance acceleration modes have been respectively tested by digital simulation and compared with the conventional complementary filter proposed in [14]. As switching proportional and integral coefficients in the complementary filter is similar to adjusting the noise matrix *R* explained in Section 2.4, the comparison is mainly between the proposed filter (Proposed) and the complementary filter (CF) in the digital and kinematic vehicle experiments. In the digital simulation experiments, the gyroscope and accelerometer error parameters can be set as shown in Table 1. The vehicle accelerates in the X axis on the body frame (front-right-down), which mainly influences the pitch estimate precision. In Figure 3a, Figure 4a and Figure 5a, the disturbance acceleration estimate is compared with the ideal disturbance acceleration in the X axis on the body frame and the time response for adaptively switching *c_a_* is displayed in Figure 3b, Figure 4b and Figure 5b. It has been shown that the X axis disturbance acceleration on the body frame is estimated exactly by switching *c_a_* adaptively. The time response for the pitch estimate comparison between the proposed filter (Proposed) and the complementary filter (CF) is plotted in Figure 3c, Figure 4c and Figure 5c. Moreover, the pitch estimate maximum error as well as root-mean-square (RMS) error are compared in Table 2. As revealed by simulations, the proposed method is better than the complementary filter in non-, vibration- and sustained-acceleration mode, especially in sustained-acceleration mode.

### 3.2. Kinematic Vehicle Experiments and Results

**Figure 6:**
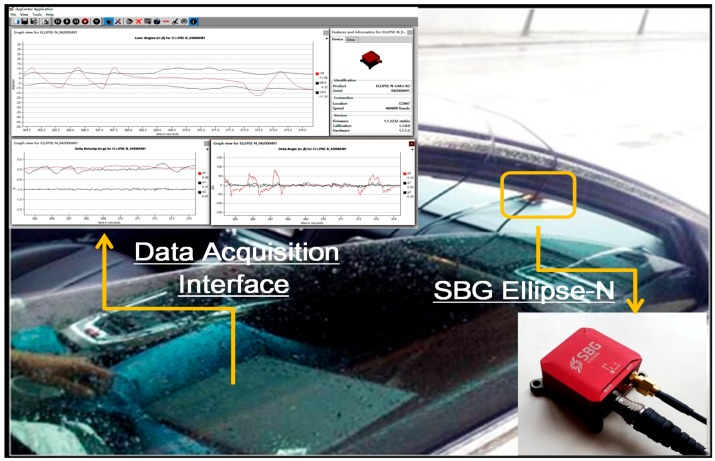
Data acquisition diagram in the kinematic vehicle experiment.

**Figure 7:**
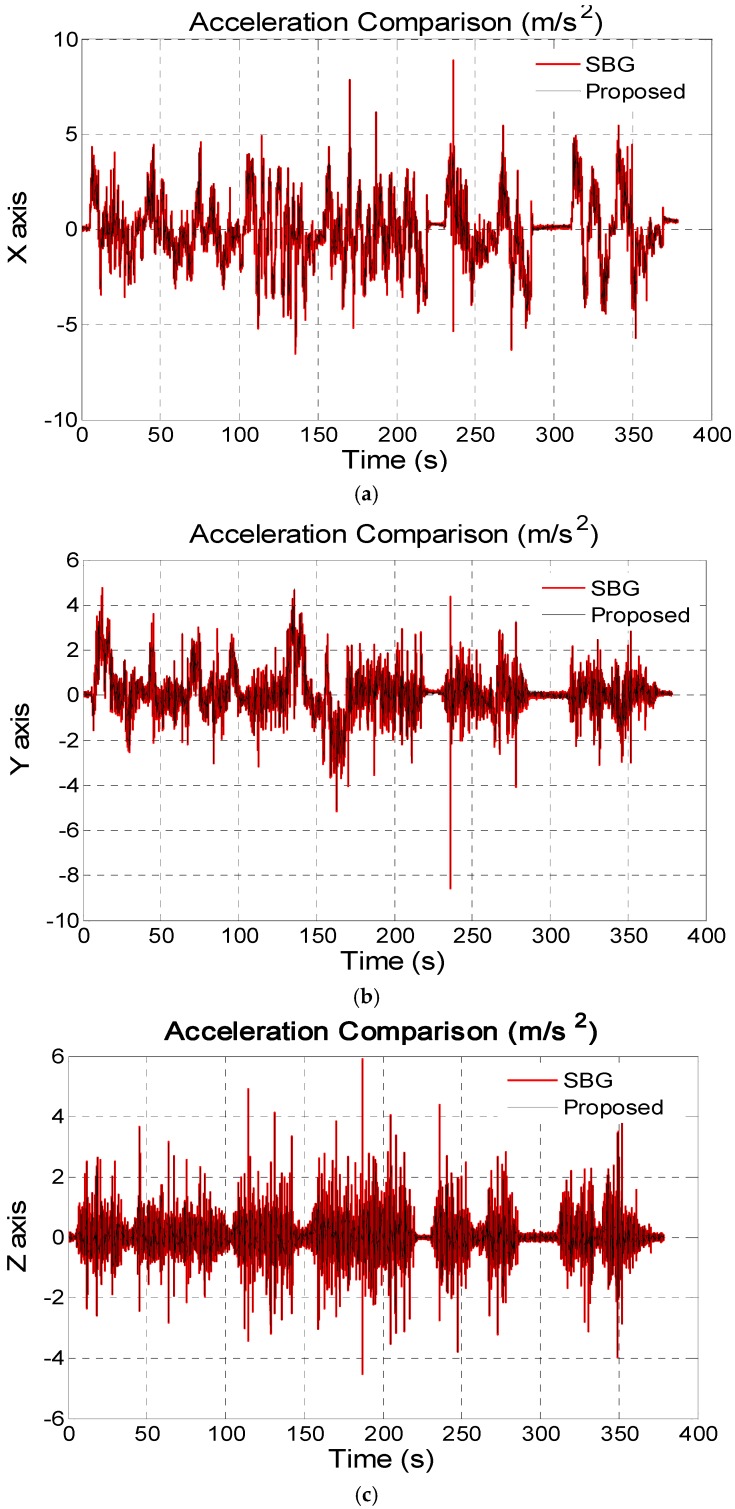
(**a**) X axis disturbance acceleration estimation on body frame in the kinematic vehicle experiment; (**b**) Y axis disturbance acceleration estimation on body frame in the kinematic vehicle experiment; (**c**) Z axis disturbance acceleration estimation on body frame in the kinematic vehicle experiment.

**Figure 8:**
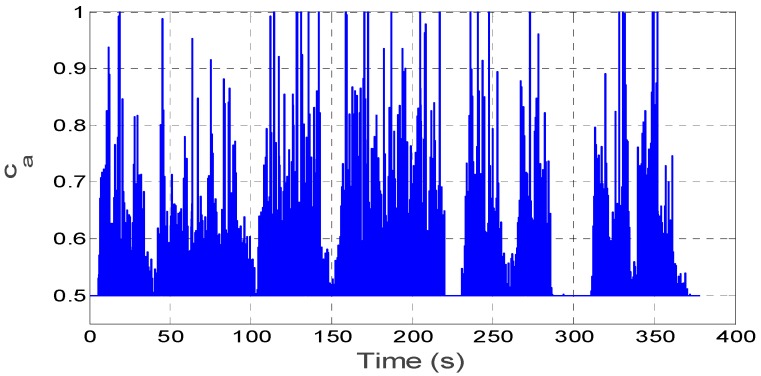
Adaptive switch for *c_a_* in the kinematic vehicle experiment.

**Figure 9:**
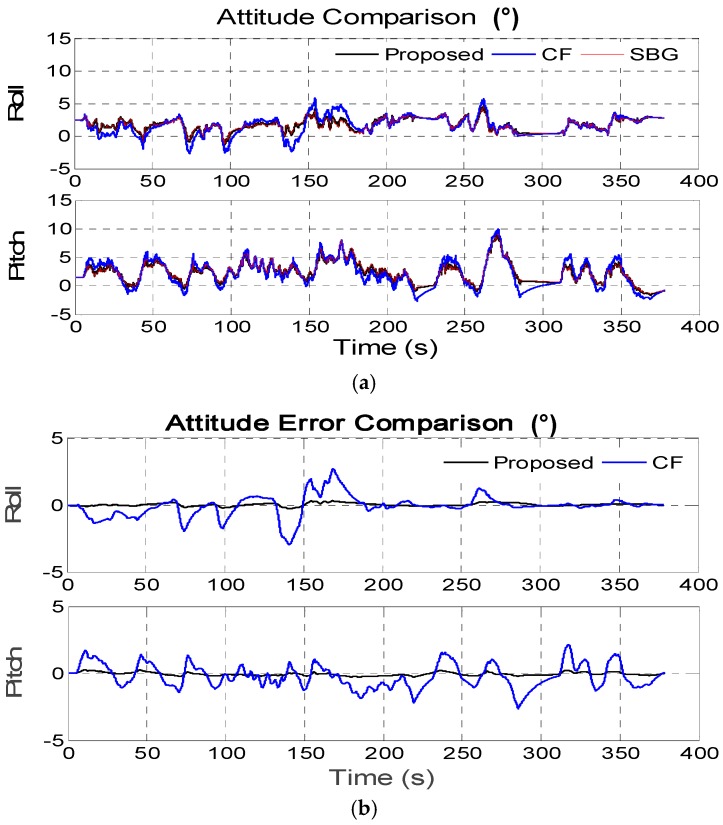
(**a**) Roll and pitch estimate comparison in the kinematic vehicle experiment; (**b**) Roll and pitch estimate error comparison in the kinematic vehicle experiment.

In this section, the kinematic vehicle experiment was designed and the outputs of the on-board MEMS ARS (SBG Ellipse-N shown in Figure 6) are set as standard comparison results, so as to effectively evaluate the proposed algorithm. The roll and pitch precision in the SBG Ellipse-N are both 0.2°, and MEMS gyroscope and accelerometer performance indexes can be seen in Table 3. In the dynamic kinematic vehicle experiment, we also utilize the outputs of the MEMS gyroscopes and accelerometers from the SBG Ellipse-N as sensor data sources to validate the advantages of the proposed algorithm. In the approximately 400 s-long experiment, the vehicle uninterruptedly accelerated and decelerated as seen from Figure 7a–c and the filter decay coefficient *c_a_* is adaptively switched as shown in Figure 8. Figure 7a–c shows that the disturbance acceleration could be estimated effectively and adaptively. Additionally, the roll and pitch estimate precision based on the proposed approach is higher than that of the complementary filter shown in Figure 9 and Table 4. Compared with the outputs of SBG Ellipse-N, Table 4 shows that the maximum estimate error of roll and pitch is approximately 0.3° and the RMS error of roll and pitch is approximately 0.1° using the proposed approach.

## 4. Conclusions

This paper has considered the problem of the influence of the disturbance acceleration on the roll and pitch estimate in MEMS ARS and put forward a disturbance acceleration adaptive estimate and correction approach. The proposed approach estimates the disturbance acceleration error in the nine state vectors Kalman filter, and adaptively estimates and corrects the disturbance acceleration combining with switching the filter decay coefficient. Even though the rule of the threshold value for selecting the ranges to identify acceleration mode is obtained experimentally based on the characteristics of accelerometers, it is still possible to make further performance improvements by tuning the ranges and adjusting the filter decay coefficient *c_a_* in accordance with different vehicles and different acceleration characteristics. In this paper, the digital simulation and the kinematic vehicle experiment have demonstrated the good estimation characteristics of the disturbance acceleration. In the kinematic vehicle experiments with uninterruptedly accelerated and decelerated conditions, the algorithm gave a maximum error of 0.3° and RMS error of 0.1° in roll and pitch compared to the outputs of a high-precision SBG Ellipse-N, which is far better than the attitude estimation performance based on the complementary filter. The presented approach offers several advantages over other implementations:

1. A disturbance acceleration estimation model based on a first-order Markov process is set up and the disturbance acceleration estimation error is estimated in the Kalman filter.
2. In the acceleration estimate model, it analyses the relationship of the filter decay coefficient *c_a_* and the correlation time *τ* of a first-order Markov process, which is the theoretical reason for switching *c_a_*.
3. The switching logic for *c_a_* is designed in accordance with different disturbance acceleration modes.

Owing to these advantages, the disturbance acceleration is estimated and corrected accurately and adaptively. Thus, the disturbance acceleration influence on the attitude estimate is isolated effectively, which thereby improves the attitude estimate precision in ARS. Since the estimation results of digital simulations and the kinematic vehicle experiment are encouraging, a possible idea for future research is that the next experimental work will be tested in other vehicles (e.g., UAVs) so as to further verify the advantages of the proposed approach.

## Figures and Tables

**Table 1:** MEMS gyroscope and accelerometer error parameters for the digital simulation.

Error Parameters		Gyroscope	Accelerometer
**First-order Gauss-Markov** **Process**	**Correlation Time**	100 s	-
	**White Noise**	10 °/h	-
**White Noise**		0.1 °/s	5 mg

**Table 2:** Max and RMS pitch error in three acceleration modes.

Pitch Error (°)	Non-Acceleration		Vibration-Acceleration		Sustained-Acceleration	
	Proposed	CF	Proposed	CF	Proposed	CF
**MAX**	1.65 × 10^−^^2^	5.95 × 10^−2^	4.63 × 10^−1^	1.28	8.44 × 10^−1^	2.98
**RMS**	3.18 × 10^−2^	1.26 × 10^−1^	1.95 × 10^−1^	6.12 × 10^−1^	5.32 × 10^−1^	2.12

**Table 3:** MEMS gyroscope and accelerometer performance index of the SBG Ellipse-N.

Performance Index	Gyroscope	Acceleration
**Bias Instability**	8 °/h	10 mg
**Angle Random Walk**	$0.18{}^{\circ}/\sqrt{h}$	-
**Speed Random Walk**	-	$200{}^{\circ}\mu g/\sqrt{h}(X,Y)$ $300{}^{\circ}\mu g/\sqrt{h}(Z)$

**Table 4:** Max and RMS of roll and pitch error in the kinematic vehicle experiment.

	Roll Error (°)		Pitch Error (°)	
	Proposed	CF	Proposed	CF
**MAX**	3.24 × 10^−1^	2.96	2.89 × 10^−1^	2.65
**RMS**	1.14 × 10^−1^	8.09 × 10^−1^	1.31 × 10^−1^	9.12 × 10^−1^
